# Prognostic value of perioperative serum low-density lipoprotein cholesterol level for postoperative prognosis of pancreatic cancer: a retrospective study

**DOI:** 10.1186/s12944-023-01851-x

**Published:** 2023-06-30

**Authors:** Hanxuan Wang, Yulin Li, Jincan Huang, Youwei Ma, Shaocheng Lyu, Ren Lang

**Affiliations:** Hepatobiliary Surgery Department, Beijing Chao-Yang Hospital, Beijing, No. 8 Gongti South Road, Chao-Yang District, 100020 China

**Keywords:** Pancreatic cancer, Low-density lipoprotein cholesterol, Postoperative, Tumour recurrence, Long-term prognosis

## Abstract

**Background:**

As a common malignant tumour, pancreatic cancer (PC) has the worst clinical outcome. Early evaluation of the postoperative prognosis has certain clinical value. Low-density lipoprotein cholesterol (LDL-c), which is mainly composed of cholesteryl esters, phospholipids, and proteins, plays an important role in transporting cholesterol into peripheral tissues. LDL-c has also been reported to be correlated with the occurrence and progression of malignant tumours and can predict postoperative prognosis in various tumours.

**Aims:**

To determine correlation between serum LDL-c level and clinical outcome in PC patients after surgery.

**Methods:**

Data of PC patients that received surgery at our department from January 2015 to December 2021 were retrospectively analysed. Receiver operating characteristic (ROC) curves between perioperative serum LDL-c at different timepoints and survival rate at postoperative 1-year were drawn, and the optimal *cut-off* value was calculated. Patients were categorized into low and high LDL-c groups, and their clinical data and outcome were compared. Univariate and multivariate analyses were applied to screen out risk markers for poor prognosis of PC patients after surgery.

**Results:**

The area under the ROC curve of serum LDL-c at 4 weeks after surgery and prognosis was 0.669 (*95% CI*: 0.581–0.757), and the optimal *cut-off* value was 1.515 mmol/L. The median disease-free survival (DFS) rates of low and high LDL-c groups were 9 months and 16 months, respectively, and the 1-, 2- and 3-year DFS rates were 42.6%, 21.1% and 11.7% in low LDL-c group, respectively, and, 60.2%, 35.3% and 26.2% in high LDL-c group, respectively (*P* = 0.005). The median overall survival (OS) rates of low and high LDL-c groups were 12 months and 22 months, respectively, and the 1-, 2- and 3-year OS rates were 46.8%, 22.6% and 15.8% in low LDL-c group, respectively, and 77.9%, 46.8% and 30.4% in high LDL-c group, respectively (*P* = 0.004). Multivariate analysis confirmed low postoperative 4-week serum LDL-c as independent risk marker for early tumour recrudesce and poor clinical outcome in PC patients.

**Conclusion:**

High postoperative 4-week serum LDL-c is a prognostic marker for prolonged DFS and OS time in PC patients.

## Introduction

Pancreatic cancer (PC) is a digestive system malignant tumour with increasing incidence [1]. The American Cancer Association estimates that there will be 64,050 cases of newly diagnosed PC in the US in 2024, accounting for only 3% of all estimated new cancer cases. However, 8% of all expected new tumour-related deaths are caused by PC, ranking fourth among all malignant tumours [2]. Its 5-year survival rate is only 11% [2]. Currently, surgical treatment is the possible cure for PC, but the postoperative outcome is still unsatisfactory. Only about 30% of patients can survive 5 years even after radical surgery [3, 4]. Thus it is valuable to effectively predict the clinical outcome of PC patients at early postoperative stage in clinical practice.

Low-density lipoprotein cholesterol (LDL-c) mainly originates from very low density lipoprotein cholesterol (VLDL-c) and is transported to peripheral organs and tissues through the blood stream [5]. Serum LDL-c is taken up under the mediation of low-density lipoprotein receptor to provide exogenous cholesterol for cells [6, 7]. Since LDL-c is mainly derived from food intake and liver synthesis, serum LDL-c can be affected by the nutritional status of patients and has been reported to be an indicator of malnutrition. Das et al. discovered a declined in serum LDL-c among malnourished people compared to healthy controls [8]. Hrnciarikova et al. discovered that LDL-c and prealbumin level were linearly related and confirmed LDL-c as a novel marker for malnutrition in elderly patients [9]. Due to recent in-depth research on tumour metabolic reprogramming, more attention has been devoted to the correlation between LDL-c and various tumours. High serum LDL-c was confirmed as a risk marker for tumorigenesis of breast cancer and can also promote the its proliferating and metastatic capacity by reducing intercellular adhesion [10, 11]. Prostate cancer is also a steroid-targeted tumour; although the relationship between its occurrence and serum LDL-c level remains controversial, it has been confirmed that LDL-c is able to improve proliferating, invasive and metastatic ability of prostate cancer [12–14]. In addition, serum LDL-c levels have also been reported to have prognostic value for postoperative prognosis in malignant cancer. For ampullary adenocarcinoma and ovarian cancer patients, the recurrence-free survival (RFS) rate significantly improved in high serum LDL-c patients compared to low serum LDL-c patients, which was partly attributed to the metabolic reprogramming of cancer cells [15, 16]. In terms of PC, its tumorigenesis and progression are also correlated with LDL-c. High preoperative serum LDL-c is considered as a risk of PC tumorigenesis and can promote the progression and metastasis of PC through the STAT3 pathway, indicating potential value of LDL-c in predicting clinical outcome of PC patients [13, 17]. Considering the strong relevance between LDL-c and PC, it is hypothesized that the serum LDL-c level during perioperative period may be able to predict the clinical outcome of PC patients after surgery. Current researches mainly center on the relationship of LDL-c with tumorigenesis and progression of PC, paying little attention to connection of perioperative serum LDL-c with the postoperative outcomes in PC patients. This is the first research reporting the effect of serum LDL-c in predicting the clinical outcome of postoperative PC patients.

Current research mainly focuses on investigating the correlation between serum LDL-c at perioperative period and clinical outcome in postoperative PC patients, revealing its potential prognostic effect.

## Methods

### **Patient screening**

**Fig. 1:**
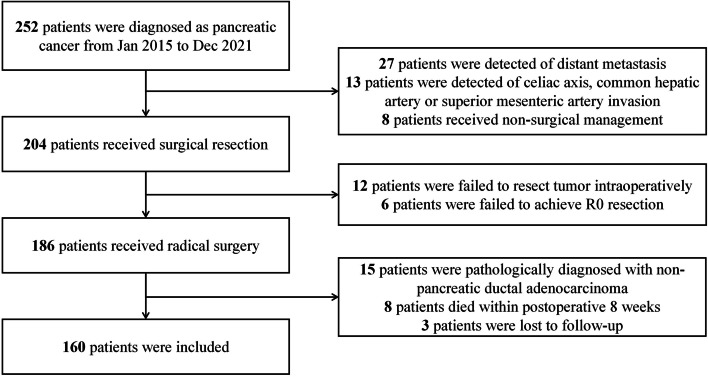
Patients screening

PC patients who underwent surgery at Hepatobiliary Surgery Department, Beijing Chao-yang Hospital, from Jan 2015 to Dec 2021 were retrospectively analysed and were further included or excluded base on the inclusion and exclusion criteria shown in following paragraph (Fig. 1).

The following were the inclusion criteria: (1) PC patients that received surgeries in our department from January 2015 to December 2021; (2) no requirement for age and sex; (3) no contraindications of operation in assesment before surgery; (4) successful resection of tumor during operation; (5) pathological examination after operation indicated pancreatic ductal adenocarcinoma and intraoperative R0 resection; and (6) integrity of follow-up data.

The following were the exclusion criteria: (1) Detected of invasion to important abdominal artery or distant metastasis intraoperatively; (2) unable to achieve R0 resection intraoperatively; (3) application of lipid-lowering drugs during perioperative period; and (4) postoperative survival time less than 2 months.

All surgical plan and therapeutic schedule were informed consent from included individuals and their family, and the Ethnic Committee of Beijing Chao-yang Hospital (No. 2020-D.-302) granted application of their clinical data.

### Patient grouping

Serum LDL-c was assessed at 5 time points, including preoperation, postoperative 3 days, postoperative 1 week, postoperative 4 weeks and 8 postoperative 8 weeks. Preoperative serum LDL-c level was assessed on the second day after admission. Blood samples (3.5 ml) were collected from included patients after overnight (12 h) fasting and centrifuged within 2 h after acquisition to isolate the serum. Then, LDL-c levels were measured using direct measurement (Siemens Healthcare Diagnostics Inc. USA). The normal range of serum LDL-c was < 3.30 mmol/L. Receiver operating characteristic (ROC) curves between serum LDL-c levels at different timepoints and survival condition at postoperative 1 year was obtained and the area under the curve (AUC) and optimal *cut-off* value were ascertained. Based on the *cut-off* value, included patients were categorized to low and high LDL-c group.

### Data analysis and follow-up strategy

Perioperative data from records of included patients were compared within these two groups in current research. Follow-up was first scheduled at 1 month and 3 months postoperatively. Then, follow-up interval was set at once every 3 months in postoperative 2 years and every 6 months afterward till tumour recrudesce or mortality. Data including results of blood examinations (Routine examination, biochemistry, carbohydrate antigen 19–9), imageological evaluation (abdominal and pulmonary enhanced computerized tomography), current treatment regimen, postoperative tumour recrudesce and long-term prognosis were collected at every follow-up and further compared within different groups.

### Statistical analysis

Means ± standard deviation were used to present normal distributed quantitative data, and median (interquartile range) were applied to present non-normal distritbuted quantitative data. T tests were utilized to compare normal distributed data and rank sum test was adopted in comparing non-normal distributed data. Fisher’s test was utilized to compare enumeration data under the condition of theoretical frequency < 1 or sample size < 40; otherwise, chi-square test was utilized. Clinical outcome was calculated with Kaplan–Meier method and and compared with log-rank test. Univariate analysis was first utilized to screeen out variables with statistic significance and then Cox regression analysis was utilized in determining risk markors. *P* values < 0.05 indicated statistic significance. SPSS (version 26.0; IBM Corporation, US) was applied in statistical analysis.

## Results

### General condition of included patients

This study involved 160 patients, among which 89 were males and 71 were females. Male–female ratio was 1.3: 1. The included patients had an average age of 63.0 ± 10.1 years old. In terms of the intial symptoms, 61 patients showed abdominal pain, 73 patients showed jaundice, 8 patients showed atypical digestive symptoms, and the remaining18 patients were asymptomic and found in health examination. 53 individuals (33.1%) a history of diabetes. Jaundice reduction treatment was arranged in 36 of 73 patients with jaundice before surgery,, among which 32 patients received percutaneous transhepatobiliary drainage and the rest 4 patients underwent endoscopic retrograde cholangiopancreatography.

### Perioperative condition

Twelve patients received neoadjuvant chemotherapy in current research. Surgery of included patients went smooth and achieved en bloc excision. Venous excision and reconstruction was proceed for patients with suspectable portal venous system invasion. The intraoperative hemorrhage volume of the included patients was 500 (400, 800) ml. 62 of 160 individuals (38.8%) had intraoperative transfusions. Operative duration was 10 (8, 12) h.

Diagnosis of pancreatic ductal adenocarcinoma and negative surgical margin were pathologically confirmed in included individuals. 47 (29.3%), 101 (63.1%) and 12 (7.5%) patients were diagnosed as low, moderate and high differentiation PC, respectively.. The tumour diameter of the included patients was 3.5 (2.5, 4.5) cm. Lymphatic metastasis was confirmed by pathology in 100 patients (62.5%).

Postoperative complications were observed in 48 patients (32.0%). comprising pancreatic fistula (*n* = 20), intraperitoneal infection (*n* = 15), delayed gastric emptying (*n* = 15), intraperitoneal bleeding (*n* = 2), gastrointestinal bleeding (*n* = 3), pneumonia (*n* = 2), biliary fistula (*n* = 1), pulmonary embolism (*n* = 1) and portal venous system thrombosis (*n* = 1). Patients were hospitalized for 19 (15, 25) days after surgery.

#### Overall survival condition

**Fig. 2:**
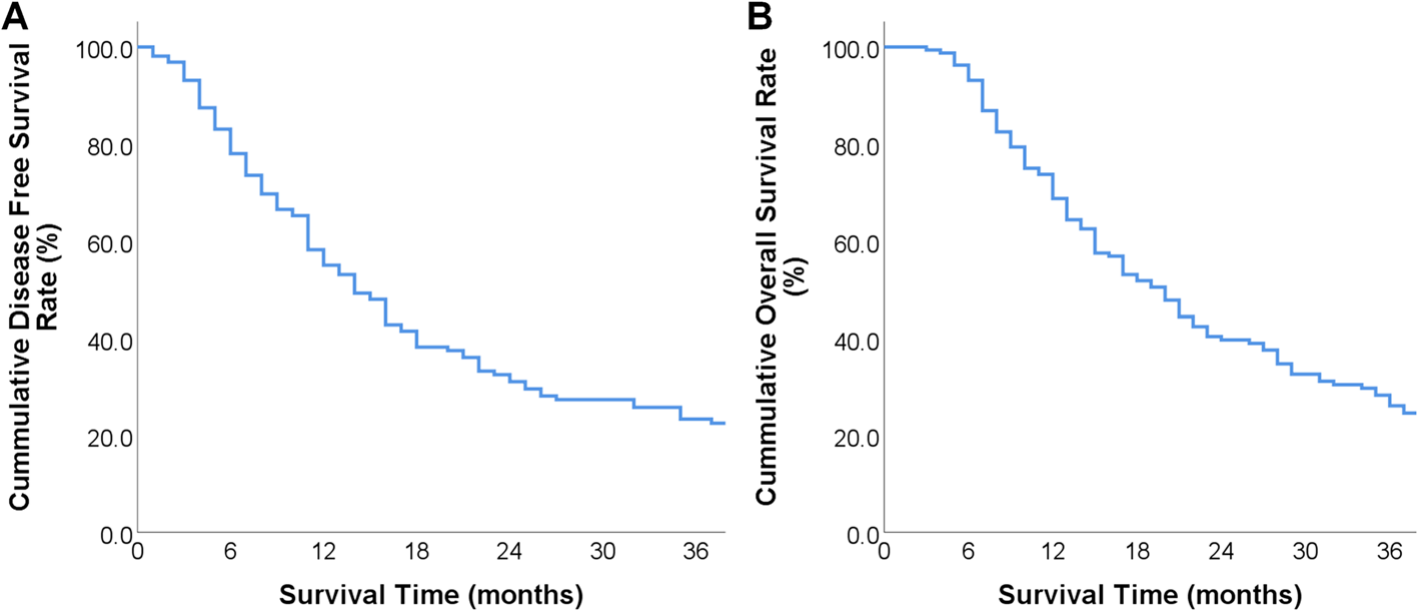
Clinical outcome of included individuals: **A**: DFS condition of included individuals; **B**: OS condition in included individuals

Patients were followed up until Dec 2022. 86 patients (53.8%) received adjuvant chemotherapy after surgery and the chemotherapeutic cycle range from 1 to 18. Included individuals had a median DFS time and overall survival (OS) time of 14 months and 20 months, and 1-year, 2-years and 3-years DFS rate and OS rate after surgery were 55.0%, 31.1%, 22.5%,and 68.7%, 39.7%, 26.1%, respectively (Fig. 2).

#### Grouping condition

**Fig. 3:**
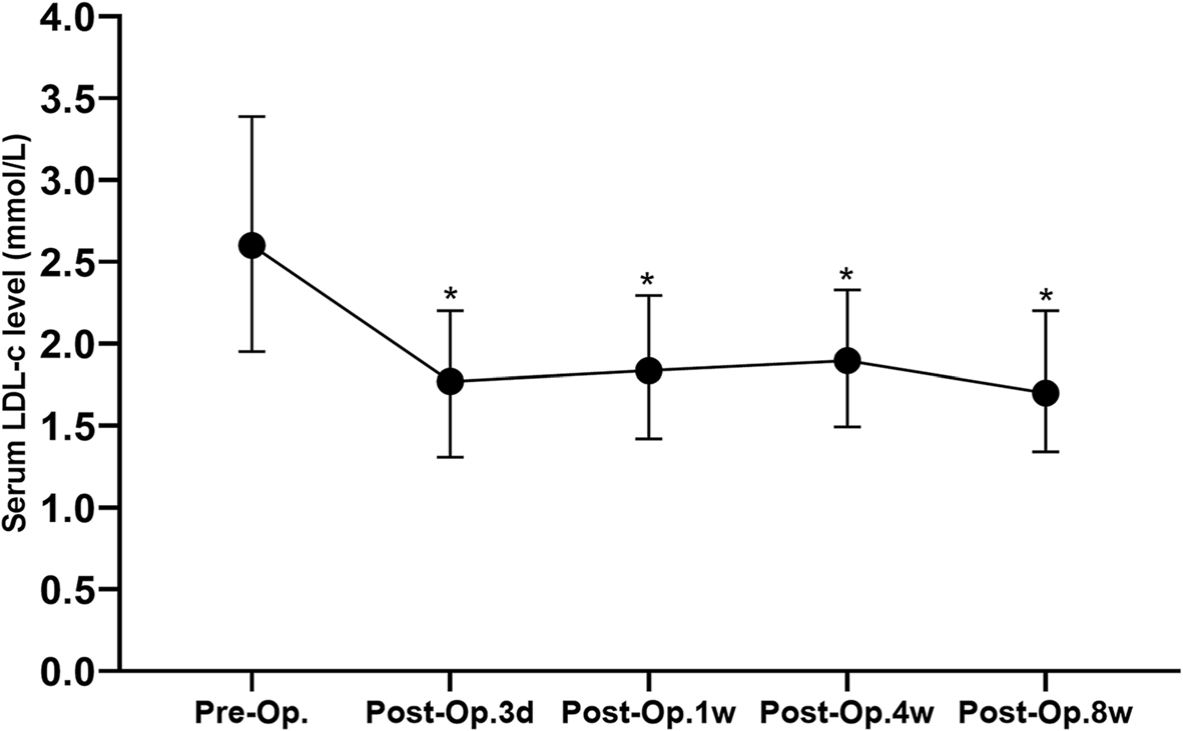
Perioperative change of serum LDL-c levels (*: Compared with preoperative serum LDL-c level, *P* < 0.05; Pre-op.: Preoperation; Post-op.: Postoperative)

**Fig. 4:**
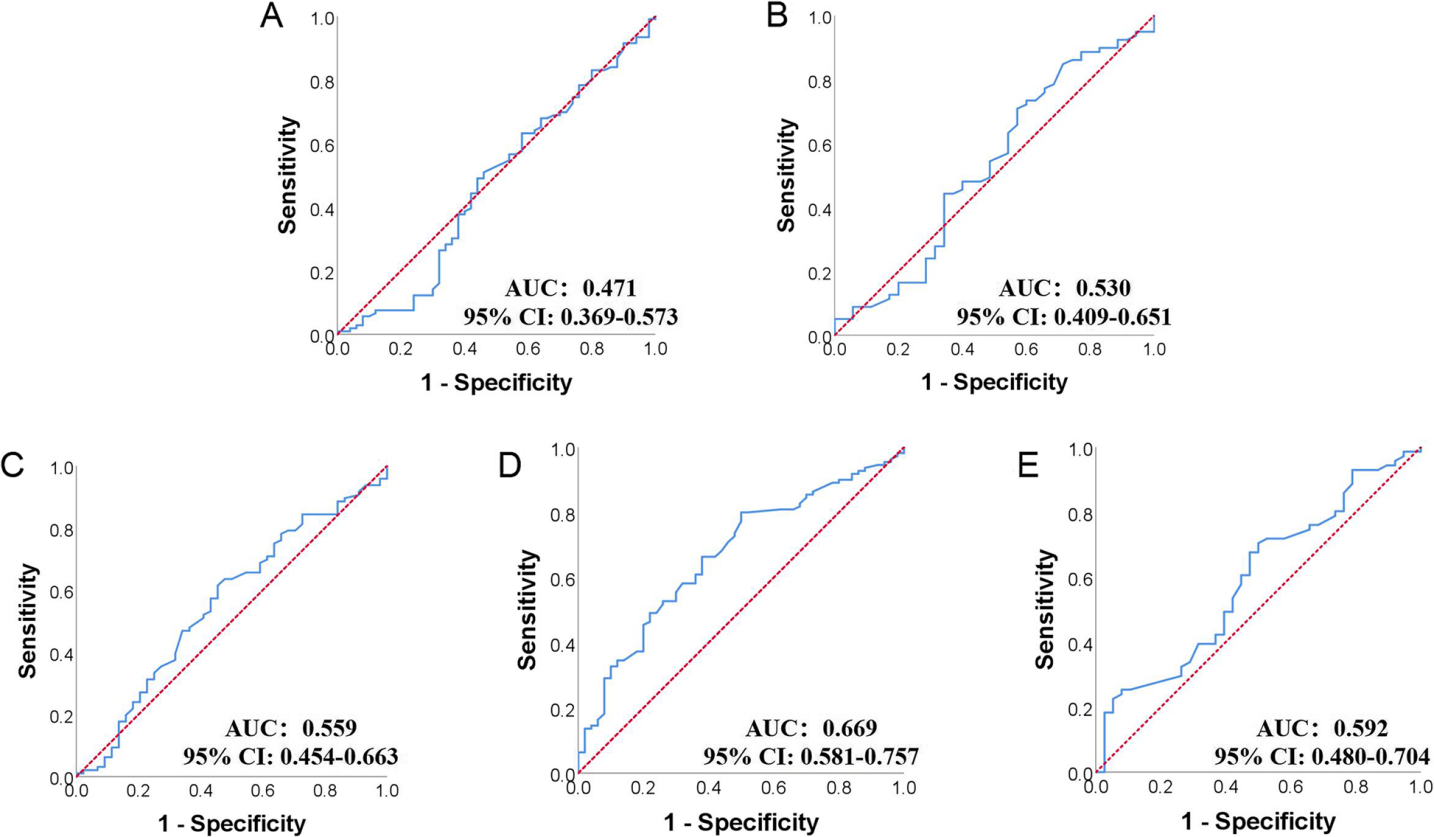
ROC curve of serum LDL-c at different timepoints and survival condition at 1 year after surgery in PC patients: A: ROC curve between serum LDL-c level at preoperation and survival rate at postoperative 1 year; B: ROC curve between serum LDL-c level at postoperative 3 day and survival rate at postoperative 1 year; C: ROC curve between serum LDL-c level at postoperative 1 week and survival rate at postoperative 1 year; D: ROC curve between serum LDL-c level at postoperative 4 weeks and survival rate at postoperative 1 year; E: ROC curve between serum LDL-c level at postoperative 8 weeks and survival rate at postoperative 1 year

Variation of serum LDL-c in included individuals during perioperative period is shown in Fig. 3. Decreasing serum LDL-c was discovered at early postoperative period. Then it recovered afterwards, reaching a peak at postoperative 4-week. Later on the serum LDL-c level gradually declined. Serum LDL-c levels were significantly lower than preoperative serum LDL-c levels at every postoperative time point. As shown in Fig. 4, ROC curves between serum LDL-c levels at preoperation, postoperative 3 days, postoperative 1 weeks, postoperative 4 weeks, postoperative 8 weeks and survival condition at postoperative 1 year were drawn and the AUC values were 0.471, 0.530, 0.559, 0.669 and 0.592. Postoperative 4-week serum LDL-c was determined to be study object because of its AUC and variation tendency of perioperative LDL-c level. The *cut-off* value of serum LDL-c level at postoperative 4 weeks was calculated to be 1.515 mmol/L with a 80.0% sensitivity and 50.0% specificity in predicting the survival condition at postoperative 1 year. Patients were further categorized into low LDL-c group (postoperative 4-week serum LDL-c ≤ 1.515 mmol/L, n = 47) and high LDL-c group (postoperative 4-week serum LDL-c > 1.515 mmol/L, n = 113) according to the *cut-off* value.

#### Intragroup comparison of general data and long-term clinical outcome

**Fig. 5:**
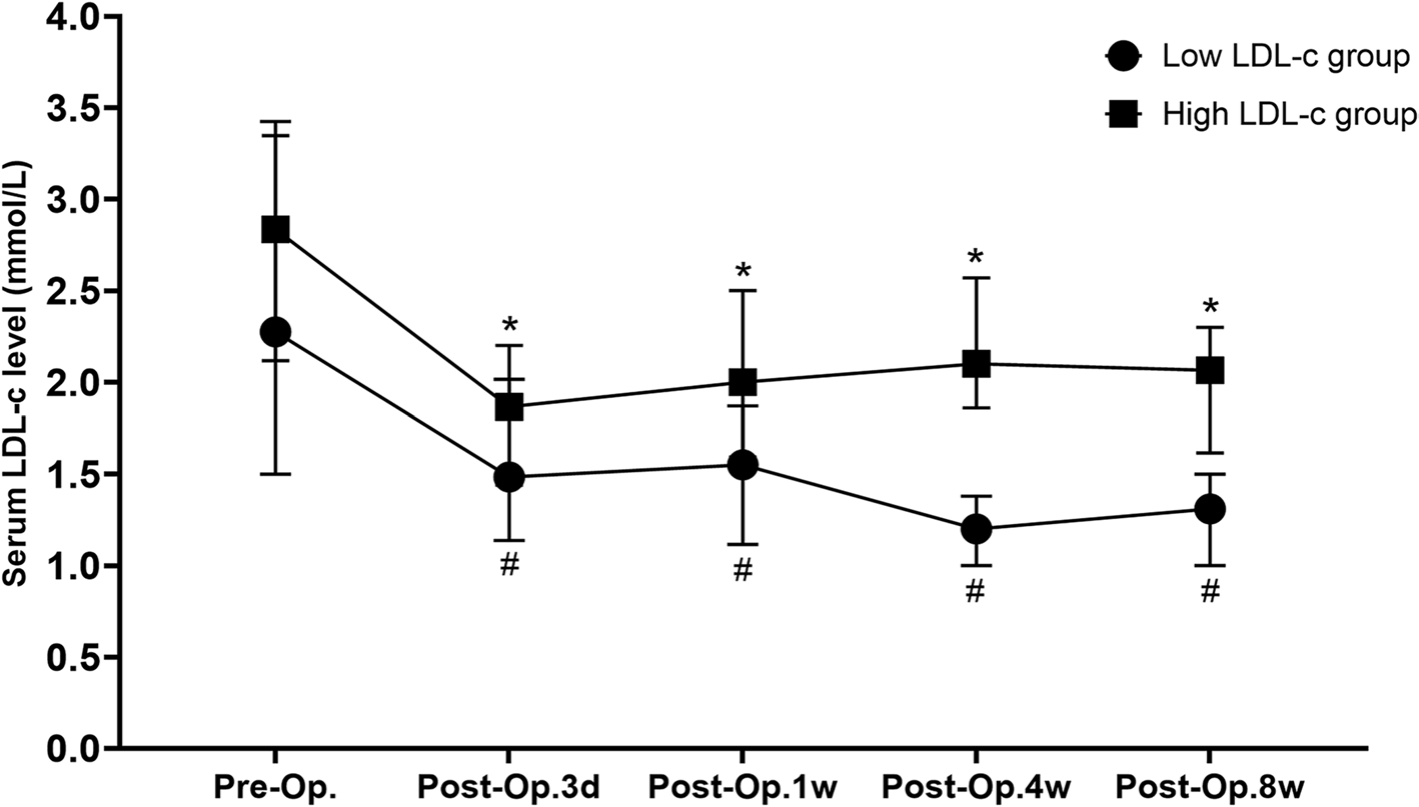
Perioperative change of serum LDL-c level in different groups (*: In comparison with baseline serum LDL-c before surgery in high LDL-c group, *P* < 0.05; #: In comparison with baseline serum LDL-c level before surgery in low LDL-c group, P < 0.05, Pre-op.: Preoperation, Post-op.: Postoperation)

Perioperative data of low and high LDL-c group was compared and shown in Table 1, illustrating a significantly higher serum LDL-c levels at post-operative 3-day, 1-week, 4-week and 8-week in high LDL-c group compared to low LDL-c group (*P* < 0.05). Change of serum LDL-c levels in perioperative period in these two groups were displayed in Fig. 5, showing that serum LDL-c started recovering at 1 week after the temperate decrease in early stage after surgery and reached a plateau at postoperative 4 weeks in high LDL-c group. However, serum LDL-c level continued to decrease after surgery until 4 weeks postoperatively, at which point the serum LDL-c level started to recover in low LDL-c group. In both groups, postoperative serum LDL-c levels at different postoperative time points decreased obviously compared with preoperative baseline (*P* < 0.005). Major postoperative complications were compared within low and high LDL-c group (Table 2), showing no statistic difference in the postoperative fatality rate and complications incidence within these two groups (*P* > 0.05)

**Table 1:** Perioperative information of included individuals within low and high LDL-c group

Variables	Low LDL-c group (*n* = 47)	High LDL-c group (*n* = 113)	*P* Value
Sex (Male/Female)	26/21	63/50	0.960
Age (Years)	61.7 ± 11.7	63.7 ± 9.5	0.262
Diabetes (Yes/No)	18/29	35/78	0.370
Preoperative jaundice reduction treatment (Yes/No)	10/37	26/87	0.811
Preoperative total bilirubin (μmol/L)	41.2 (14.0, 85.7)	40.5 (10.4, 136.7)	0.891
Preoperative carbohydrate antigen 19–9 (U/ml)	215.8 (59.8, 1745.5)	182.9 (33.1, 567.6)	0.242
Preoperative albumin (g/L)	36.7 ± 5.2	36.6 ± 5.1	0.923
Preoperative LDL-c (mmol/L)	2.20 (1.49, 3.36)	2.81 (2.11, 3.33)	0.024
Postoperative 3 days LDL-c (mmol/L)	1.49 (1.14, 2.02)	1.86 (1.44, 2.25)	0.005
Postoperative 1 week LDL-c (mmol/L)	1.54 ± 0.56	2.16 ± 0.69	0.000
Postoperative 8 weeks LDL-c (mmol/L)	1.31 (1.00, 1.50)	2.07 (1.62, 2.30)	0.000
Postoperative 4 weeks albumin (g/L)	34.6 ± 5.6	36.4 ± 5.0	0.040
Postoperative BMI	22.97 ± 2.59	23.41 ± 2,98	0.260
Postoperative 4 weeks BMI	22.08 ± 2.62	22.82 ± 2.96	0.118
Postoperative 4 weeks TG (mmol/L)	1.33 (1.08, 1.79)	1.20 (0.98, 1.53)	0.045
Intraoperative hemorrhage (ml)	500 (400, 800)	500 (400, 800)	0.102
Intraoperative transfusion (Yes/No)	22/25	40/73	0.177
Operation time (hours)	10 (8, 12)	9 (7.5, 11)	0.116
Tumour size(cm)	3.5 (2.5, 4.0)	3.5 (2.5, 4.5)	0.577
Tumour differentiation (Low/Moderate-high)	15/32	32/81	0.649
Portal venous system invasion (Yes/No)	23/24	47/66	0.229
Adjuvant chemotherapy (Yes/No)	3/44	9/104	0.987
Lymphatic metastasis (Yes/No)	32/15	68/45	0.347
Postoperative chemotherapy (Yes/No)	23/24	63/50	0.431

**Table 2:** Major complications after surgery in the low and high LDL-c group

Variations	Low LDL-c group (*n* = 47)	High LDL-c group (*n* = 113)	*P* Value
Length of hospital stay after surgery(days)	18 (15, 23)	19 (14, 27)	0.973
Postoperative complications	15	33	0.733
Biochemical fistula	2	7	0.914
Pancreatic fistula			
Grade B pancreatic fistula	2	5	1.000
Grade C pancreatic fistula	2	2	0.718
Delayed gastric emptying	4	11	1.000
Abdominal infection	5	7	0.521
Intra-abdominal haemorrhage	1	1	1.000
Gastrointestinal haemorrhage	0	3	0.626

**Fig. 6:**
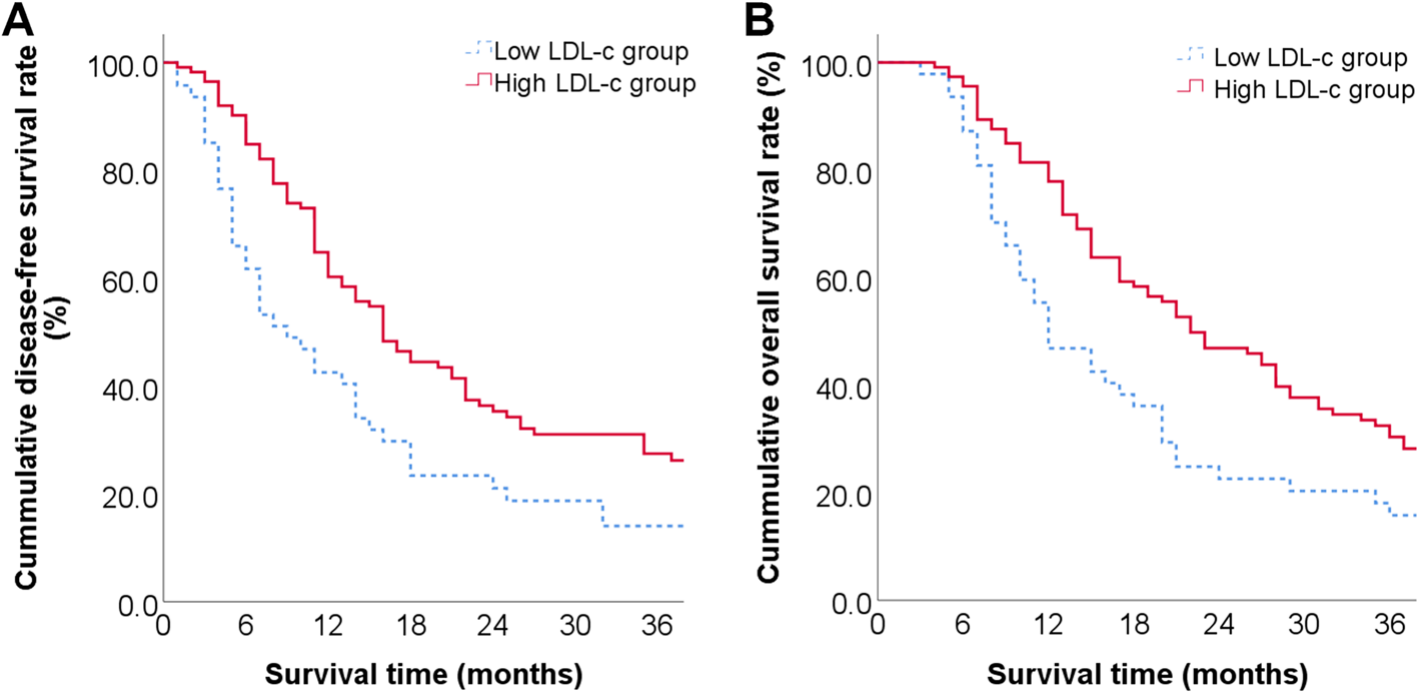
Clinical outcomes of included patients in different groups: A: DFS condition within low and high LDL-c group; B: OS condition within low and high LDL-c group

Patients had median DFS times of 9 months and 16 months in low and high LDL-c groups. The DFS rates at postoperative 1 year, 2 years and 3 years were 42.6%, 21.1% and 11.7%, 60.2%, 35.3% and 26.2% (*P* = 0.005, Fig. 6-A). Patients in low and high LDL-c group had median OS times of 12 months and 22 months.. The OS rates at postoperative 1 year, 2 years and 3 years were 46.8%, 22.6% and 15.8%, 77.9%, 46.8% and 30.4% (*P* = 0.005, Fig. 6-B).

#### Analysis of risk markers for tumour recrudesce after radical surgery in PC patients

In univariate analysis, tumour recrudesce was defined as a dependent variable while preoperative, intraoperative and postoperative clinical information were defined to be independent variables. According to results, postoperative 4-week serum LDL-c level, tumour differentiation degree, tumour size and lymphatic metastasis were possible hazard markers for tumour recrudesce after surgery (Table 3). Cox regression analysis including these 4 markers were applied for further multivariate analysis, confirming postoperative 4-week serum LDL-c level, tumour differentiation degree and lymphatic metastasis as independent risk markers for tumour recrudesce after surgery in PC patients (Table 4). Patients with higher serum LDL-c level at postoperative 4 weeks, better tumour differentiation degree and free of lymph node metastasis have less risk of tumour recrudesce after surgery.

**Table 3:** Univariate analysis of tumour recrudesce after surgery in included individuals

Variations	Cases (*n* = 160)	1-year DFS rate (%)	3-year DFS rate (%)	*χ*^2^ Value	*P* Value
Sex				0.660	0.416
Male	89	47.3	20.4		
Female	71	64.5	25.2		
Age (years)				0.015	0.904
≤ 60	60	59.5	18.5		
> 60	100	52.3	25.1		
Diabetes				0.045	0.833
Yes	53	49.4	22.2		
No	107	57.7	22.8		
Jaundice reduction treatment before surgery				0.000	0.994
Yes	36	47.2	18.3		
No	124	57.3	22.7		
Preoperative total bilirubin (μmol/L)				0.094	0.760
≤ 21	70	57.4	23.9		
> 21	90	53.3	20.2		
Preoperative carbohydrate antigen 19–9 (U/ml)				0.044	0.834
≤ 37	40	52.5	24.2		
> 37	120	55.9	20.9		
Preoperative albumin (g/L)				3.574	0.059
≤ 40	119	51.5	18.7		
> 40	41	65.2	30.7		
Postoperative 4 weeks albumin (g/L)				3.367	0.067
≤ 40	124	50.7	19.7		
> 40	36	69.4	32.2		
Pre-operative BMI				5.000	0.082
< 18.5	11	72.7	43.6		
18.5–23.9	87	49.3	19.3		
≥ 24.0	62	59.7	20.4		
Postoperative 4 weeks BMI				2.835	0.242
< 18.5	13	61.5	34.6		
18.5–23.9	96	50.9	18.3		
≥ 24.0	51	60.8	22.3		
Preoperative LDL-c (mmol/L)				1.085	0.297
≤ 2.375	64	51.1	22.3		
> 2.375	96	57.6	24.2		
Postoperative 3 days LDL-c (mmol/L)				0.515	0.473
≤ 1.460	61	49.5	20.8		
> 1.460	99	57.3	23.9		
Postoperative 1 week LDL-c (mmol/L)				1.206	0.272
≤ 1.785	71	42.3	17.7		
> 1.785	89	64.9	23.4		
Postoperative 4 weeks LDL-c (mmol/L)				7.903	0.005
≤ 1.515	47	40.4	11.7		
> 1.515	113	60.2	26.2		
Postoperative 8 weeks LDL-c (mmol/L)				2.519	0.113
≤ 1.505	55	46.3	12.3		
> 1.505	105	55.6	18.4		
Operation time (hours)				1.373	0.241
≤ 8	58	57.5	33.7		
> 8	102	53.5	18.1		
Intraoperative hemorrhage volume (ml)				1.058	0.304
≤ 800	90	58.9	25.2		
> 800	70	49.9	19.0		
Intraoperative transfusion				1.829	0.176
Yes	62	47.4	16.0		
No	98	59.8	27.5		
Tumour differentiation degree				18.268	0.000
Low	47	29.8	7.2		
Moderate/High	113	65.7	29.1		
Tumour size (cm)				7.440	0.006
≤ 4	112	58.5	27.5		
> 4	48	44.3	6.6		
Lymphatic metastasis				12.105	0.001
Yes	100	44.6	14.8		
No	60	72.7	35.5		
Portal venous system invasion				1.889	0.169
Yes	70	51.4	17.3		
No	90	57.8	26.9		
Postoperative complications				0.793	0.373
Yes	48	44.8	21.5		
No	112	59.4	22.9		
Postoperative chemotherapy				0.005	0.945
Yes	86	59.8	20.0		
No	74	49.4	23.2		

#### Analysis of risk markers for 1-year survival condition after surgery in PC patients

In univariate analysis, long-term survival condition was defined as a dependent variable, and the preoperative, intraoperative and postoperative clinical information were defined to be independent variables. According to results, postoperative 4-week serum LDL-c level, tumour differentiation degree, tumour size and lymphatic metastasis were possible risk markers for 1-year survival condtion after surgery (Table 5). Cox regression analysis including these 4 markers were applied for further multivariate analysis, confirming postoperative 4-week serum LDL-c level, tumour differentiation degree and lymphatic metastasis as independent risk markers for poor 1-year survival condition after surgery (Table 6). Patients with higher serum LDL-c level at postoperatve 4 weeks, better tumour differentiation degree and free of lymphatic metastasis indicated better long-term postoperative survival condition.

**Table 4:** Cox regression analysis of tumour recrudesce after surgery in included individuals

Variations	*RR* Value	95% *Confidence interval*	*P* Value
Postoperative 4-week LDL-c	0.632	0.438–0.912	0.014
Degree of tumour differentiation	2.103	1.428–3.095	0.000
Tumour size	1.342	0.887–2.029	0.163
Lymphatic metastasis	1.881	1.291–2.740	0.001

**Table 5:** Univariate analysis of 1-year survival condition after surgery in included individuals

Variations	Case (*n* = 160)	1-year OS rate (%)	3-year OS rate (%)	*χ*^2^ Value	*P* Value
Sex				0.335	0.563
Male	89	66.3	25.4		
Female	71	71.8	26.9		
Age (hours)				0.070	0.792
≤ 60	60	71.7	19.5		
> 60	100	67.0	30.5		
Diabetes				0.361	0.548
Yes	53	67.9	28.8		
No	107	69.2	24.9		
Jaundice reduction treatment before surgery				0.497	0.481
Yes	36	55.6	20.7		
No	124	72.6	26.6		
Preoperative total bilirubin (μmol/L)				0.020	0.887
≤ 21	70	72.9	31.4		
> 21	90	65.6	26.0		
Preoperative carbohydrate antigen 19–9 (U/ml)				0.268	0.605
≤ 37	40	70.0	32.1		
> 37	120	68.3	22.4		
Preoperative albumin (g/L)				2.255	0.133
≤ 40	119	67.2	23.5		
> 40	41	73.2	34.5		
Postoperative 4 weeks albumin (g/L)				2.031	0.154
≤ 40	124	66.1	23.8		
> 40	36	77.8	25.5		
Pre-operative BMI				4.517	0.105
< 18.5	11	81.8	42.4		
18.5–23.9	87	64.4	20.6		
≥ 24.0	62	72.6	29.4		
Postoperative 4 weeks BMI				2.782	0.249
< 18.5	13	69.2	32.3		
18.5–23.9	96	65.6	20.8		
≥ 24.0	51	74.5	32.9		
Preoperative LDL-c (mmol/L)				1.806	0.179
≤ 2.375	64	65.6	22.6		
> 2.375	96	70.8	28.5		
Postoperative 3 days LDL-c (mmol/L)				0.324	0.569
≤ 1.460	61	63.9	23.7		
> 1.460	99	71.7	26.5		
Postoperative 1 week LDL-c (mmol/L)				0.764	0.382
≤ 1.785	71	62.0	27.9		
> 1.785	89	74.2	24.8		
Postoperative 4 weeks LDL-c (mmol/L)				8.323	0.004
≤ 1.515	47	46.8	15.8		
> 1.515	113	77.9	30.4		
Postoperative 8 weeks LDL-c (mmol/L)				2.670	0.102
≤ 1.505	55	52.5	17.1		
> 1.505	105	72.5	21.6		
Operation time (hours)				1.932	0.165
≤ 8	58	75.9	36.6		
> 8	102	64.9	20.8		
Intraoperative hemorrhage volume (ml)				0.912	0.340
≤ 800	90	74.4	29.6		
> 800	70	61.4	21.6		
Intraoperative transfusion				1.997	0.158
Yes	62	62.9	17.9		
No	98	72.4	30.9		
Tmour differentiation degree				14.154	0.000
Low	47	48.9	11.4		
Moderate/High	113	77.0	32.4		
Tumour size (cm)				7.028	0.008
≤ 4	112	72.0	32.6		
> 4	48	59.5	8.1		
Lymphatic metastasis				14.425	0.000
Yes	100	62.0	17.6		
No	60	80.0	40.3		
Portal venous system invasion				3.255	0.071
Yes	70	61.4	20.7		
No	90	74.4	30.4		
Postoperative complications				0.230	0.631
Yes	48	64.6	27.1		
No	112	70.5	25.5		
Postoperative chemotherapy				0.173	0.677
Yes	86	75.6	22.4		
No	74	60.8	31.3		

**Table 6:** Cox regression analysis of postoperative 1-year survival in included individuals

Variation	*RR* Value	95% *Confidence interval*	*P* Value
Postoperative 4-week LDL-c	0.602	0.417–0.870	0.007
Degree of tumour differentiation	1.875	1.279–2.748	0.001
Tumour size	1.403	0.944–2.084	0.094
Lymphatic metastasis	1.950	1.341–2.834	0.000

## Discussion

PC is a malignant tumour in digestive system It has the 10^th^ highest incidence of all tumors and has a 0.5%–1% annual growth rate. [18, 19]. It has a 5-year survival rate of just 11.0% in US and 7.2% in China, making it the top four cause of cancer-related death and the most malignant tumour with the worst long-term prognosis worldwide [2, 20]. Despite the great progress in adjuvant therapy, radical operation remains the only feasible curative therapy for PC. Although postoperative radiotherapy and chemotherapy have been proven to further increase the DFS rate and OS rate of patients, the survival rate at postoperative 5 years is still only 30%, far from satisfying [3, 4, 21]. There is a certain clinical value in effectively predicting the long-term clinical outcome of PC patients at early postoperative period to guide postoperative re-examination and treatment.

LDL-c is mainly composed of cholesteryl ester, triglyceride and phospholipids. Cholesteryl ester and triglyceride constitute the core of LDL-c, and their exterior is coated with phospholipids, apolipoprotein B100 and free cholesterol [5, 22]. Serum LDL-c, which is a metabolite of VLDL-c, can be transported to various tissues and organs through the blood stream and bring exogenous cholesterol into cells by interacting with low density lipoprotein receptor located at cell surface, playing a crucial role in cholesterol transportation [6, 23]. The expression level of low density lipoprotein receptor is an important regulatory factor for serum LDL-c levels.

According to previous researches, LDL-c has a strong correlation with the occurrence of coronary artery atherosclerosis [24]. In recent years, as the abnormal lipid metabolism in tumours has been continuously revealed, LDL-c has also been found to have close connection with tumorigenesis and progression of PC, breast cancer, prostate cancer, and other malignant tumours [10, 11, 13, 17, 25]. In addition, serum LDL-c levels have also been reported to have certain prognostic effect for tumor recrudesce and survival outcome after surgery in various types of malignant tumour. According to Jung et al., the tumour recrudesce rate of low serum LDL-c patients increased by 1.87 times than that of high serum LDL-c patients in postoperative breast cancer patients. These results were attributed to a variety of possible reasons, including high serum LDL-c levels indicating better nutritional status, prompting patients to use statins, and reducing vascular endothelial growth factor expression to inhibit tumour neovascularization [26]. Li et al. discovered that higher preoperative serum LDL-c levels were correlated with longer DFS times and lower incidence of vascular invasion in ampullary carcinoma patients [15]. After comparing ovarian cancer patients with different preoperative serum LDL-c levels, Zhu et al. observed a significant higher 5-year DFS rate in high preoperative LDL-c levels patients than low serum LDL-c levels patients, which may be caused by the excessive uptake of LDL-c to satisfy the increasing cholesterol need of ovarian tumour cells [16]. At present, no relevant research on relation of perioperative LDL-c levels and clinical outcome in PC patients has yet been published. In this study, it was found that the postoperative 4-week serum LDL-c level has a close correlation with long-term clinical outcome in postoperative PC patients. This was the first research reporting low serum LDL-c level as a risk marker independent of tumour differentiation degree and lymphatic metastasis for poor postoperative DFS and OS rates in PC patients, thus confirming the potential predicting value of perioperative LDL-c levels in PC. However, inconsistent with other tumours, preoperative serum LDL-c levels had no clinical value in predicting long-term outcome after surgery in included individuals. Zhou et al. retrospectively analysed small cell lung cancer patients and discovered that preoperative serum LDL-c levels had predictive value only for limited-stage tumours, which may be related to changes in the tumour metabolic microenvironment [27]. In current research, 70 patients had portal vein invasion, and 100 patients had lymphatic metastasis, indicating late tumour stage and large tumour burden in the included patients. The excessive consumption of serum LDL-c caused by advanced tumours led to a relatively low serum LDL-c level in both groups, which may be the potential cause of this result. Further studies are needed to verify this finding.

The pancreas has important physiological function in digestion and glucose regulation. Over 50% of PC patients are undernourished at diagnosis due to the invasion and damage of tumours. Furthermore, surgical treatment of PC is always time-consuming and traumatic, and pancreaticoduodenectomy and total pancreatectomy require digestive tract reconstruction to restore its continuity, making malnutrition a common perioperative complication in PC patients [28, 29]. Das et al. discovered an obvious decline in serum LDL-c levels among malnourished individuals, showing the close correlation between serum LDL-c levels and nutritional status[8]. Reduced VLDL-c synthesis in liver caused by insufficient substrate secondary to malnutrition may be the potential reason. Therefore, low postoperative 4-week serum LDL-c levels may indicate malnutrition in patients, and the relatively low postoperative 4 weeks serum albumin level further confirmed the relatively poor nutritional status in the low LDL-c group, supporting our hypothesis. According to recent studies, postoperative malnutrition serves as an independent risk marker for poor prognosis after pancreatic surgery. Jin et al. and Shi et al. performed studies in patients who received pancreaticoduodenectomy and total pancreatectomy and found that patients with postoperative malnutrition not only had a higher incidence of complications after surgery but also had a significantly lower DFS time and OS time than those without malnutrition [30, 31]. This may be one of the potential reasons why serum LDL-c levels have prognostic effect for long-term clinical outcome in PC patients after surgery.

As an important component of the cell membrane, cholesterol participates in various physiological functions, and certain levels of intracellular cholesterol is crucial for immune cells to maintain its function. Appropriate serum LDL-c levels also take part in maintaining antitumour effects of immune cells since LDL-c is the main source of exogenous cholesterol for cells. In addition, recent studies have found that LDL-c itself also has a certain effect on cellular immunity. Newton et al. observed the effect of LDL-c in promoting the transformation from juvenile T cells into Th1 cells, a subtype of helper T cells associated with the natural killer cells and CD8^+^ T cells activation [32]. Babl et al. further reported that high LDL-c levels could not only prompt the transformation from CD4^+^ T cells into central memory T cells with stronger and longer antitumour ability and upregulate the level of CD4^+^T-cell costimulatory factor CD40 ligand but also increase the antitumour effect of anti-PD-1 immunity [33]. A higher postoperative 4-week serum LDL-c level may indicate better immune cell function and a better response to further antitumour comprehensive treatment, which may be one of the possible mechanisms of its predictive value.

In this research, serum LDL-c levels at preoperation, postoperative 3 days, postoperative 1 week, postoperative 4 weeks and postoperative 8 weeks were included to reflect the variation of serum LDL-c levels during perioperativ period. It was found that serum LDL-c levels in PC patients declined transiently at the early postoperative stage, which may be caused by surgical consumption as well as the imbalance of cholesterol supply and demand in early stage after surgery. With the recovery of patients' body function and nutritional status after surgery and the reduction in tumour-associated LDL-c consumption, the serum LDL-c level gradually increased. At 4 weeks after surgery, patients had largely recovered from the surgical trauma and tumour consumption, causing the relatively high serum LDL-c level at that time [34]. The serum LDL-c level decreased again at 8 weeks postoperatively, which may be related to tumour recurrence and the application of postoperative adjuvant therapies.

It was also found that the DFS rate of low postoperative 4-week serum LDL-c levels patients were lower than that of high LDL-c levels patients, further confirming postoperative 4-week LDL-c level to be an independent risk marker for postoperative recurrence of PC. It is reported that tumours are often combined with a "lipid-lowering effect", which has also been reported in PC [35–37]. Sah et al. retrospectively analysed the changes in blood lipid profiles in PC patients before diagnosis and found that patients already developed significant LDL-c reduction accompanied by subcutaneous fat loss at 6–18 months before diagnosis [38]. For patients who achieved R0 resection intraoperatively, LDL-c level at postoperative 4 weeks was less affected by surgical and tumourous factors. Low serum LDL-c levels at this time may be an early manifestation before tumour recrudesce. In addition, although R0 resection is achieved in certain patients and confirmed by postoperative pathological examination, some lymph nodes and adipose tissues may still have minimal residual lesions. Low postoperative 4-week serum LDL-c levels may be a result of high LDL-c uptake by residual lesions, indicating postoperative residual tumour. This may be a potential mechanism for postoperative 4-week serum LDL-c levels to predict postoperative recrudesce of PC. Further studies are necessary to further verify the findings of current research.

In addition to the potential value of postoperative LDL-c level in predicting prognosis of PC patients, it was also found in current research that poor tumour differentiation and positive lymph node were risk markers for early tumour recrudesce and poor postoperative long-term clinical outcome in PC patients, in accordance with previous reports [39, 40]. This research further demonstrated their predictive effect for the postoperative prognosis of PC.

### Strengthens and limitations

The strengthens and clinical relevance of current research are as follow. First, this is the first research reporting the predictive effect of serum LDL-c levels on long-term clinical outcome of PC patients after surgery, thus providing a novel marker for prognostic evalutiaon at early postoperative stage. In the future, this finding also indicated the possibility of integrating serum LDL-c levels in comprehensive models to predict the outcome of PC patients more accurately. Second, since serum LDL-c level is an indicator of nutritional status, the correlation between serum LDL-c level at postoperative 4 weeks and poor prognosis reported in this research further reveals the importance of maintaining good nutritional status in PC patients at 4 weeks after surgery. Although majority of patients resume oral feeding at that time, food intake may not meet the energy requirement in some patients and can cause malnutrition, which is easy to neglect since most patients are discharged from the hospital at that time. However, this research confirmed that malnutrition at 4 weeks postoperatively can also cause poor long-term prognosis, thus emphasizing the importance of proceeding nutritional screening and necessary nutritional support at an early stage after surgery. Low serum LDL-c may also serve as a potential indicator for oral nutritional support in these patients, which requires further study for verification. Third, nutritional status can influence the tolerance of postoperative adjuvant antitumour treatment. Since the serum LDL-c level has been confirmed to be a prognostic marker for tumour recrudesce and long-term prognosis and can reflect the nutritional status, the postoperative 4-week serum LDL-c level may be an effective marker to guide the selection of comprehensive antitumour treatment in the future, thus further improving the postoperative prognosis of PC patients.

There are also certain limitations in this research. Firstly, this is a retrospective study in single centre, and further prospective studies are necessary to verify these conclusions. Secondly, current study only explored the value of serum LDL-c level in predicting postoperative recrudesce as well as clinical outcome of PC without illustrating its underlying mechanism. Thirdly, the potential guiding effect of postoperative serum LDL-c level on postoperative antitumour comprehensive treatment in PC patients was not further explored in this study, which may be a potential direction for further research.

## Conclusion

In conclusion, the peri-operative serum LDL-c level decreased transiently at the early postoperative stage and gradually increased afterwards until 4 weeks postoperatively before it decreased again according to this research. Postoperative serum LDL-c levels remained lower than preoperative levels up to 8 weeks after surgery. It was also found that high serum LDL-c levels patients at postoperative 4 weeks had prolonged DFS times and OS times compared to those with low serum LDL-c, identifying postoperative 4-week serum LDL-c level as an independent risk marker as well as a potential prognostic indicator for long-term clinical outcome of postoperative PC patients. The ability of the postoperative 4-week serum LDL-c level to reflect postoperative nutritional status, affect postoperative immune function and suggest postoperative residual tumour and early recurrence may be potential reasons for these findings in this research. This research not only provided novel prognostic markers for PC patients at the early postoperative period but also emphasized the importance of nutritional screening and necessary nutritional support at postoperative 4 weeks to improve long-term prognosis. It also provides a potential possibility to adopt serum LDL-c levels as an indicator to provide postoperative nutritional support and guide further antitumour therapy.

## Data Availability

Corresponding author will provide datasets used and/or analysed in this research upon reasonable request.

## Abbreviations

LDL-c: Low density lipoprotein cholesterol
VLDL-c: Very low density lipoprotein cholesterol
PC: Pancreatic cancer
AUC: Area under the curve
DFS: Disease-free survival
OS: Overall survival
Pre-op.: Pre-operation
Post-op.: Post-operation
RR: Relative risk
PD-1: Programmed cell death protein 1
